# Targeting of MAPK–ERK1/2 signaling by metformin in asthma: an updated mechanistic insight

**DOI:** 10.1007/s00210-026-05265-1

**Published:** 2026-04-07

**Authors:** Manal Ewaiss Hassan, Hayder M. Al-kuraishy, Athanasios Alexiou, Marios Papadakis, Safaa A. Faheem, Eman K. Rashwan, Gaber El-Saber Batiha

**Affiliations:** 1 Biochemical Unit, Pathology department, Medical College, Jouf University, Sakaka, 72388 Saudi Arabia; 2 Department of Clinical Pharmacology and Medicine, College of Medicine, Al-Mustansiriya University, Baghdad, Iraq; 3 European Academy of Sciences and Arts, Vienna, Austria; 4 University Centre for Research & Development, Chandigarh-Ludhiana Highway, Mohali, Punjab India; 5 Medical Department, Faculty of Life Sciences, University of Thessaly, 3 Panepistimiou Str., Viopolis, 41500 Larissa, Greece; 6 Department of Pharmacology and Toxicology, Faculty of Pharmacy, Egyptian Russian University, Cairo-Suez Road, Badr City, Cairo, 11829 Egypt; 7 Department of Physiology, College of Medicine, Jouf University, Sakaka, 42421 Saudi Arabia; 8 Department of Pharmacology and Therapeutics, Faculty of Veterinary Medicine, Damanhur University, Damanhur , AlBeheira, 22511 Egypt

**Keywords:** Asthma pathogenesis, MAPK/ERK1/2 signaling, Airway remodeling, Kinase-targeted therapy, AMP-activated protein kinase (AMPK)

## Abstract

Asthma is a heterogeneous chronic inflammatory airway disease characterized by persistent inflammation, dysregulated immune signaling, and progressive airway remodeling. It has been shown that asthma pathogenesis involves multiple signaling pathways. It has been illustrated that the mitogen-activated protein kinase/extracellular signal-regulated kinase 1/2 (MAPK/ERK1/2) cascade, which emerges as a central regulator linking immune activation, epithelial dysfunction, and structural remodeling of the airway, is implicated in the pathogenesis of asthma. Sustained ERK1/2 activation is consistently demonstrated in human asthmatic airways and experimental models, correlating with disease severity, inflammatory cell infiltration, and corticosteroid resistance. This review provides a comprehensive and critical discussion of the ERK1/2 signaling in asthma, spanning immune cells, airway epithelium, and airway smooth muscle. We evaluate preclinical and translational evidence supporting ERK1/2 as a therapeutic target and examine emerging pharmacological strategies, including indirect pathway modulation via AMP-activated protein kinase (AMPK) activation and drug repositioning with the anti-diabetic metformin. By integrating mechanistic insights with therapeutic implications, this review positions ERK1/2 as a pivotal signaling node for precision targeting in severe, treatment-refractory asthma.

## Introduction

Asthma is a heterogeneous chronic inflammatory airway disease characterized by persistent inflammation, dysregulated immune signaling, and progressive airway remodeling (Holgate 2008). More than 300 million people worldwide have asthma, with deaths over 250,000 per year (Bousquet et al. 2007). However, asthma-associated mortality has decreased over the years due to the development of inhaled corticosteroids, which are now the primary treatment for asthma worldwide (Wijesinghe et al. 2009). Concurrently, urbanization has recently led to a substantial acceleration in cases of allergies, such as asthma, over the previous 50 years (Alfvén et al. 2006). Socioeconomic disparities can further amplify these effects, with children in minority or low-income households disproportionately burdened by asthma morbidity (Alfvén et al. 2006). Green infrastructure and technological innovations can offer mitigation opportunities for the negative effects of urbanization, but require careful implementation to address multifactorial contributors to childhood asthma (Alfvén et al. 2006). Asthma is more prevalent in children compared to other age groups (Vital signs: asthma prevalence, disease characteristics, and self-management education 2011). Childhood asthma imposes the highest disability burden, causing almost 13.8 million days of absence from school in the United States in 2013. Notably, asthma prevalence was highest between 2001 and 2010, but decreased slightly between 2010 and 2017 (Akinbami et al. 2012). Moreover, children with asthma need to receive psychological support because asthma can lead to a lower education level and early dropout from school. More than 100 million additional asthmatic patients are expected by 2025. The global mortality of patients with asthma is a significant concern (Masoli et al. 2004). Therefore, analysis of the burden of asthma can help formulate public health policies, allocate resources, and prevent asthma. Several variables are known to affect asthma prevalence, including sex, racial and ethnic distributions, poverty rates, and location within the country (Masoli et al. 2004).

The pathogenesis of asthma is complex and involves various cellular and molecular alterations that can be replicated in a humanized mouse model. Eosinophilic infiltration is the most prominent feature of airway inflammation and is considered a multicellular process in asthma pathogenesis (Kay 2005). Eosinophilic infiltration, together with neutrophils, CD4+ T lymphocytes, and mast cells, contributes to airway inflammation and bronchial hyperresponsiveness (Kay 2005). It has been shown that both intraepithelial and subepithelial eosinophils are linked with type 2 (T2) inflammation, by increasing the expression of IL-33 and IL-5 which additively increased cysteinyl leukotriene (CysLT) production by eosinophils in individuals with and without asthma and related these findings to airway hyperresponsiveness and features of airway inflammation (Kay 2005) suggesting that intraepithelial eosinophils are associated with endogenous hyperresponsiveness and T2 inflammation and may interact with intraepithelial mast cells *via* CysLTs to regulate airway inflammation. Even in asthmatic patients who develop frequent comorbidities such as chronic rhinitis, sinusitis, atopic dermatitis, and certain food allergies, this Th2 cell-mediated inflammation is observed, given its prevalence in chronic allergic inflammation across multiple sites (Kay 2001). However, the exact role of each cell type in comorbid conditions remains debated; yet, at least among asthmatic patients with an allergic component, a clear pattern is beginning to emerge. The immunopathogenesis of allergic asthma involves coordinated interactions between innate and adaptive immune cells, leading to IgE-mediated mast cell activation, eosinophilic inflammation, and structural airway changes (Kay 2001). Recent advances have significantly refined the understanding of type-2 (T2) inflammation in asthma. In addition to Th2 lymphocytes, group-2 innate lymphoid cells (ILC2s) have emerged as important contributors to eosinophilic airway inflammation through the production of key cytokines such as IL-5 and IL-13 in response to epithelial-derived alarmins, including interleukin-25 (IL-25), interleukin-33 (IL-33), and thymic stromal lymphopoietin (TSLP) (Sahnoon et al. 2025). These cytokines orchestrate eosinophil recruitment and activation, promote immunoglobulin E (IgE) production, and contribute to airway remodeling and mucus hypersecretion, which are hallmarks of allergic asthma. The identification of these pathways has also led to the classification of asthma into distinct inflammatory endotypes and has facilitated the development of targeted biologic therapies directed against IL-5, IL-4/IL-13 signaling, and IgE, highlighting the central role of eosinophils and T2 immune responses in asthma pathophysiology (Sahnoon et al. 2025; Pelaia et al. 2025).

**Fig. 1:**
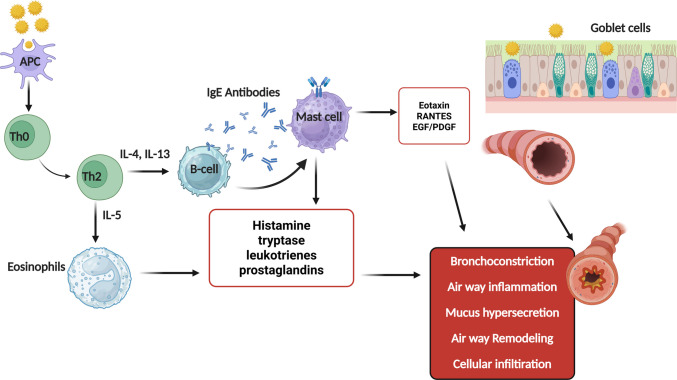
Immunopathological mechanisms underlying allergic asthma: This schematic illustrates the key immunological events driving allergic asthma. Antigen-presenting cells (APCs) activate naïve CD4⁺ T cells (Th0), promoting their differentiation into Th2 cells. Th2-derived cytokines, including IL-4 and IL-13, stimulate B cells to undergo class switching and produce IgE antibodies, while IL-5 promotes eosinophil recruitment and activation. IgE binds to high-affinity FcεRI receptors on mast cells, triggering mast cell activation upon allergen exposure and the release of inflammatory mediators, including histamine, tryptase, leukotrienes, and prostaglandins. Mast cells and eosinophils further secrete chemokines and growth factors, including eotaxin, RANTES, EGF, and PDGF, contributing to goblet cell hyperplasia, mucus overproduction, airway smooth muscle contraction, and structural remodeling. Collectively, these processes result in bronchoconstriction, airway inflammation, mucus hypersecretion, cellular infiltration, and progressive airway remodeling characteristic of asthma

The conducting airways represent the primary site of inflammation in asthma; however, with progressive disease and time, the inflammation spreads proximally and distally, involving small airways and, in certain circumstances, contiguous alveoli (Kraft et al. 1999). Contrary to the predominant submucosa inflammation observed in larger airways, the inflammation process in small airways appears to be mostly peri-airway smooth muscle in asthma (Zhang et al. 2024a). It has been shown that asthma pathogenesis involves multiple signaling pathways. Sphingosine-1-phosphate (S1P), a bioactive sphingolipid mediator, has been increasingly recognized as an important regulator of asthma pathogenesis, promoting airway smooth muscle cell proliferation, migration, and contraction, thereby contributing to airway hyperresponsiveness and remodeling in experimental asthma models (Maguire, et al. 2023). In addition, the transmembrane proteoglycan syndecan-1 can regulate numerous cell-signaling pathways in airway epithelial cells and fibroblasts in chronic asthma (Zhang et al. 2021). Furthermore, members of the tumor necrosis factor (TNF) superfamily have been implicated in asthma pathophysiology. In particular, TNF ligand superfamily member 11 (TNFSF11) and its receptor TNF receptor superfamily 11 A (TNFRSF11A) modulate transforming growth factor-β1 (TGF-β1) signaling and contribute to airway remodeling, suggesting that suppression of the TNFSF11/TNFRSF11A axis may represent a potential therapeutic strategy (D. Zhang, Zhang, Qi, et al., 2024). In addition, TNF-like cytokine 1 A (TL1A), which activates death receptor 3 (DR3), is up-regulated in asthma and promotes epithelial–mesenchymal transition (EMT) in airway epithelial cells (Zhang et al. 2024b). Collectively, these findings highlight the complex cellular and molecular signaling events underlying asthma pathogenesis (Zhang et al. 2022) **(**Fig. 1**)**.

Furthermore, the mitogen-activated protein kinase/extracellular signal-regulated kinase 1/2 (MAPK/ERK1/2) cascade, which emerges as a central regulator linking immune activation, epithelial dysfunction, and structural remodeling of the airway, is implicated in the pathogenesis of asthma (Sahnoon et al. 2025). Notably, the overactive ERK1/2 signaling pathway is correlated with asthma severity and inflammatory cell infiltration (Liu et al. 2008). In addition, adrenoceptor α2A participates in the pathogenesis of asthma by modulating the MAPK/ERK1/2 signaling pathway, making it a potential therapeutic target for asthma management (Liu et al. 2008). Consistently, administration of MEK1/2 inhibitors reduces airway inflammation and bronchial hyper-responsiveness in animal models (Duan et al. 2004; Ohnishi et al. 2009). Interestingly, the anti-diabetic metformin reduces inflammation by targeting multiple signaling pathways, including the MAPK/ERK1/2 pathway, in subjects with asthma (Ararat et al. 2024). It is clear from this substantial body of work, however, that a broader literature review is needed on the complex role of ERK1/2 across a variety of cell types (including immune cells such as T cells, epithelial cells, and airway smooth muscle cells) and in different forms of asthma. However, the exact role of the overactive ERK1/2 signaling pathway remains unclear. Thus, this review aims to provide a comprehensive overview of the ERK1/2 signaling cascade in asthma, covering the essential biology of the MAPK/ERK1/2 signaling cascade and evidence on the activation and pathologic roles of ERK1/2 in critical asthma-related cellular events, such as immune cell differentiation, cytokine secretion, and airway remodeling. In addition, this review aims to discuss and explain the potential therapeutic role of MEK1/2 inhibitors such as metformin in asthma.

## An overview of MAPK ERK1/2 signaling pathway

**Fig. 2:**
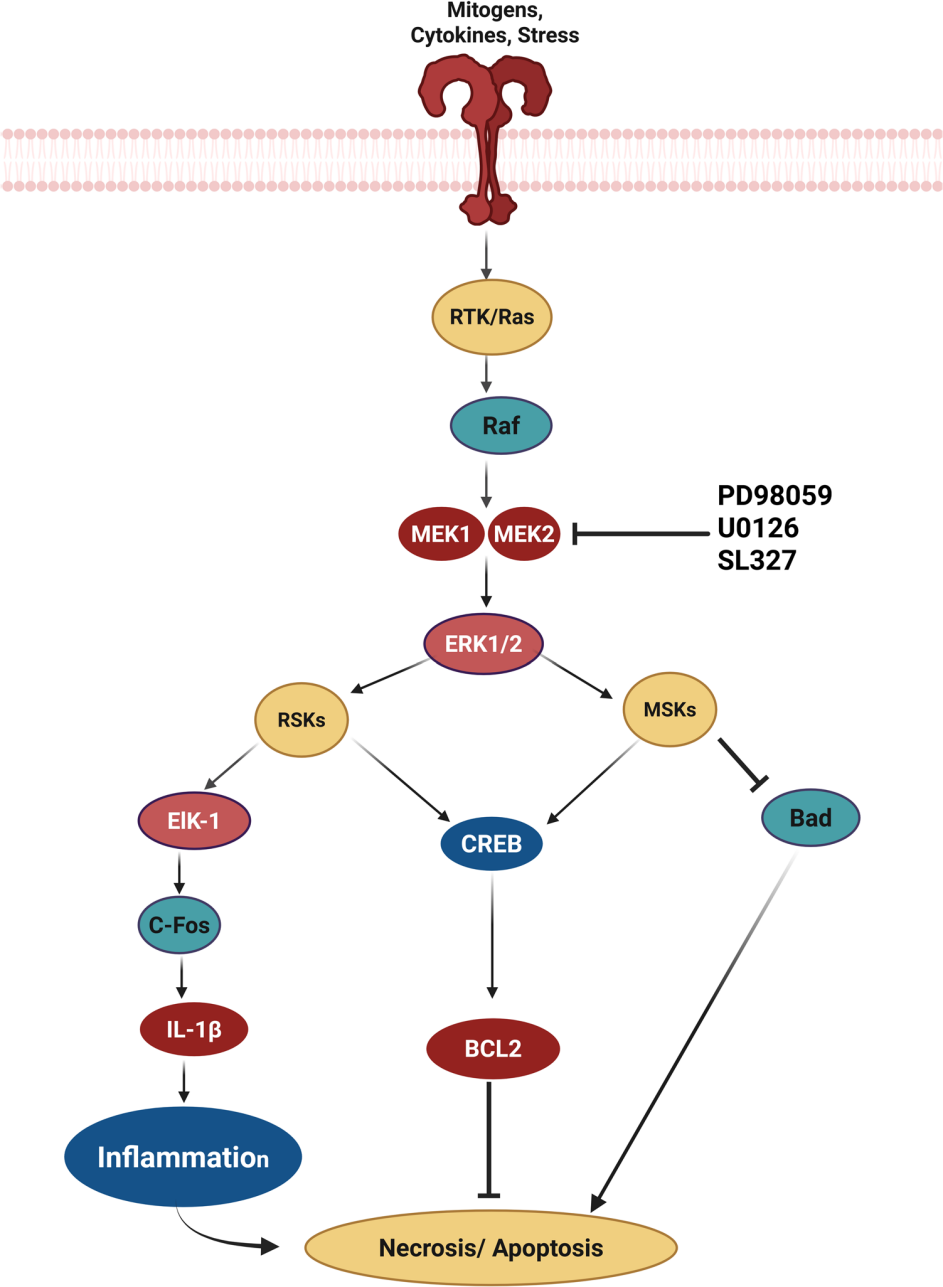
Overview of the MAPK–ERK1/2 signaling cascade and its downstream inflammatory and survival pathways: This schematic illustrates the canonical MAPK–ERK1/2 signaling pathway and its major downstream effectors. Extracellular stimuli, including mitogens, cytokines, and cellular stress, activate receptor tyrosine kinases (RTKs), leading to Ras activation and sequential signaling through Raf and MEK1/2. Activated MEK1/2 phosphorylates ERK1/2, a central signaling node regulating inflammatory responses, gene transcription, and cell fate decisions. Pharmacological MEK inhibitors, including PD98059, U0126, and SL327, block ERK1/2 activation. Downstream of ERK1/2, ribosomal S6 kinases (RSKs) and mitogen- and stress-activated kinases (MSKs) phosphorylate transcription factors such as Elk-1 and CREB. Elk-1–dependent induction of c-Fos contributes to IL-1β expression and inflammation, while CREB activation promotes BCL-2 expression and cell survival. ERK1/2 signaling also modulates apoptosis by regulating pro-apoptotic proteins such as Bad. Collectively, dysregulation of this pathway influences inflammation, necrosis, and apoptosis, highlighting its importance as a pharmacological target in inflammatory and remodeling-associated diseases

There are three major classes of MAPK, including the ERK1/2, c-Jun N-terminal kinases (JNK), and the p38 MAPK subfamilies (Chen et al. 2024). Various cellular activities, including metabolism, motility, mitosis, inflammation, differentiation, and cell death and survival, are mediated by MAPK subfamilies in response to external stimuli (Roux and Blenis 2004). Both ERK1 and ERK2 are ubiquitously expressed, with ERK2 generally expressed at slightly higher levels in most mammalian tissues, and they exhibit functional redundancy in many pathways (Lucas et al. 2022). Numerous stimuli, such as DNA damage, growth factor deprivation, cytokines, and UV irradiation, have been shown to trigger JNK and p38 MAPK activation via distinct three-tiered MAPK signal transduction pathways (Khan et al. 2024). At the intracellular level, multiple extracellular stimuli converge on the MAPK pathways, with ERK1/2 acting as a central signaling hub that regulates inflammation, gene transcription, and cell fate decisions (Park 2023). The canonical MAPK-ERK1/2 signaling cascade and its major downstream effectors are illustrated in Fig. 2.

In typical ERK1/2 pathways, ligand-induced activation of receptor tyrosine kinases (RTKs) at the plasma membrane activates and recruits the small GTPase RAS in its GTP-bound form, which then triggers dimerization and activation of MAP3K RAF to initiate a downstream phosphorylation cascade for sequential activation of MAP2K MEK and MAPK ERK (Lavoie and Therrien 2015). The three-tiered Raf/MEK/ERK cascade is activated by G-protein-coupled cell surface receptors and other receptors like the tyrosine kinase receptors with the help of different isoforms of the GTP-binding protein Ras upon activation (Kamme et al. 1995). Various isoforms of the serine/threonine kinase Raf serve as downstream targets activated by Ras (Geyer and Wittinghofer 1997). Isoforms of the dual-specificity kinases MEK1 and MEK2 are the next targets and are phosphorylated by activated Raf, and the Raf kinases, in turn, phosphorylate ERK1/2 proteins (Geyer and Wittinghofer 1997). MEK inhibitors, such as PD98059, U0126, and SL327, prevent ERK activation (Shackelford and Yeh 2006).

Importantly, activation of the ERK pathway can augment inflammation by enhancing interleukin 1β (IL-1β) expression, thereby promoting necrosis (Wang et al. 2004). ERK1/2 plays a crucial role downstream of immune receptors in promoting inflammatory gene expression in response to infection and cell or tissue damage. ERK1/2 activation plays an important role in inflammatory signaling pathways involved in innate immune responses and contributes to the regulation of pro-inflammatory cytokine expression during tissue injury and inflammatory conditions (Wang et al. 2004). In the airway tissue of asthmatic humans and asthmatic mice, ERK1/2 is activated. In a rat model of asthma, inhibition of MEK1/2, the upstream activator of ERK1/2, inhibits airway inflammation (Duan et al. 2004). Trametinib, an inhibitor of MAPK/ERK kinase (MEK) activity that possesses anti-inflammatory properties, has been permitted for clinical use (Petrini and Giaccone 2025). Trametinib suppresses inflammation in LPS-activated macrophages *in vitro* and protects against murine acute lung injury by inhibiting the MEK-ERK-Egr-1 pathway (Duan et al. 2004). Moreover, MAP3K8 is a key mediator of the early inflammatory response and may be a potential target in inflammatory diseases (Duan et al. 2004).

In addition to the foundational studies that first characterized ERK1/2 signaling in inflammatory responses, recent research has further clarified the involvement of ERK1/2 in airway inflammation and asthma pathogenesis (Cremades-Jimeno et al. 2025). ERK1/2 activation has been reported to regulate cytokine production, epithelial cell responses, airway smooth muscle proliferation, and immune cell activation in the asthmatic airway microenvironment (Makled and El-Sheakh 2023). Moreover, increasing evidence indicates that ERK1/2 signaling interacts with multiple inflammatory pathways, including MAPK subfamilies, oxidative stress signaling, and growth factor-mediated responses, thereby contributing to airway inflammation and structural remodeling in asthma. These findings support the concept that ERK1/2 functions as a central signaling node linking inflammatory stimuli with downstream transcriptional and cellular responses involved in airway inflammation (Lucas et al. 2022).

These findings suggest that the MAPK signaling pathway controls many signaling pathways in inflammatory diseases. Considering how the MAPK signaling pathway influences the inflammatory response and downstream signaling, it therefore has the potential to provide targets for a range of common inflammatory diseases.

## The role of MAPK ERK1/2 in asthma

It has been shown that the MAPK signaling pathway is intricate in airway and lung inflammation underlying asthma and chronic obstructive pulmonary disease (COPD) [Table 1] (Pelaia et al. 2021). Notably, numerous environmental agents, such as aeroallergens, cigarette smoke, and airborne pollutants, activate the MAPK p38α isoform, which, in turn, up-regulates the expression of multiple pro-inflammatory cytokines and chemokines (Pelaia et al. 2021). Consequently, p38 MAPK-induced bronchial inflammation and remodeling contribute to the development, persistence, and amplification of airflow inflammation, which is the hallmark of asthma and COPD (Pelaia et al. 2021). It has been observed that triggered p38 MAPK is crucial for IL-33-induced potentiation of TNF-α secretion from natural killer (NK) cells stimulated by IL-12 in asthma (Ochayon et al. 2020). In asthmatic patients, inhibition of neutrophil apoptosis is at least in part dependent on bronchial production of survival factors for neutrophils, such as IL-6, IL-8, and MCP-1, whose secretion can be induced by regulatory proteins S100A8 and S100A9 via activation of p38 MAPK (Kim et al. 2020). Findings from pre-clinical studies demonstrated that p38 MAPK activation and inflammatory changes increase counts of eosinophils, neutrophils, and lymphocytes, and increase levels of IL-17A in bronchoalveolar lavage fluid and in lung tissue (Jaiswal et al. 2020; Zhang et al. 2019). (Jaiswal et al., 2020; Zhang et al., 2019).

**Table 1:** Role of the MAPK signaling pathway in asthma

Studies	Findings	Ref.
*In vitro*	Triggered p38 MAPK is crucial for IL-33-induced potentiation of TNF-α secretion from natural killer (NK) cells stimulated by IL-12 in asthma.	(Ochayon et al. 2020)
*In vitro*	In an asthmatic model, S100A8 and S100A9 induce increased secretion of neutrophil survival cytokines, which are suppressed by TLR4 and AKT inhibitors.	(Kim et al. 2020)
HFD-mouse obesity	Obesity produces persistent changes in DC precursors, and the elevation of Adam17 expression is tightly coupled to p38 MAPK and is a key driver of dendritic cell proliferation.	(Jaiswal et al. 2020; Zhang et al. 2019).
OVA-challenged asthma mouse model	Ozone exposure worsened OVA-challenged airway inflammation, p38 MAPK activation, and GR downregulation in OVA-sensitized and -challenged mice, effects that IL-17A mAb effectively counteracted.	(Jaiswal et al. 2020; Zhang et al. 2019.
A prospective study	Bronchial biopsies from 11 patients with atopic asthma showed increased percentages of epithelial cells expressing phospho-p38, increased numbers of subepithelial cells expressing phospho-STAT5, and increased levels of the PI3K marker phospho-ribosomal protein S6.	[Southworth et al., 2018).
A case-control study	Significant phosphorylation of ERK1/2 and p38, and their correlation with disease severity, suggest that these signaling pathways play an important role in asthma. The ERK1/2 and p38 pathways regulate epithelial cell secretory function and proliferation.	(Liu et al. 2008).
*In vivo *	Fingolimod significantly decreased monocyte chemoattractant protein-1 (MCP-1), p-ERK, and p-P38 in lung tissues of Ova-challenged mice.	(Makled and El-Sheakh 2023).
A case-control study	AKT1, MAPK13, STAT1, and TLR4 are elevated in patients with allergic asthma compared with those with non-allergic asthma.	(Cremades-Jimeno et al. 2025; Sio. et al. 2023).
A cross-sectional genetics and epidemiological study	Involvement of a functional exonic variant of ERBB2 in asthma development via modulating the MAPK signaling cascade.	(Cremades-Jimeno et al. 2025; Sio et al. 2023).
*In vitro*	Knockdown of CSK or dislodgment of caveolin-1–bound CSK restored ERK1/2 activation in Spry2−/− T cells, suggesting an essential role for Spry2 in LCK activation and T cell function.	(Sripada A et al. 2021).
*In vitro*	ITE reduces the TNF-α-induced MMP-9 expression via the H3K9 acetylation/NF-κB/AP-1 axis, highlighting a potential mechanism for mitigating MMP-9-related inflammatory disorders.	(Bahman et al. 2026).
A case-control study	Increased p38 MAPK activation in alveolar macrophages from patients with severe asthma has been associated with reduced dexamethasone inhibition of cytokine release.	(Bhavsar et al. 2010).
BALB/c mice	The MAPK pathway might be associated with the beneficial effect of intermittent hypoxia on the attenuation of allergic response in an allergen-induced mouse model.	(Sultonov et al. 2022)
*In vitro*	Inflammation and asthma transcriptionally up-regulate the aryl hydrocarbon receptor via the p38/JNK-AP1 pathway in airway smooth muscle.	(Reza et al.^2025^)

Also, immune/inflammatory cells are targeted by extracellular stimuli that activate p38 MAPK through phosphorylation. Phospho-p38 expression is up-regulated in bronchial epithelial cells of asthmatic patients (Southworth et al. 2018). By activating p38 MAPK, TGF-β triggers apoptosis in human airway epithelial cells (Pelaia et al. 2025). Furthermore, p38 MAPK triggers structural bronchial remodeling, such as thickening of the subepithelial basement membrane, by inducing the expression of cytokines, chemokines, growth factors, and other mediators of bronchial inflammation and remodeling, including immunoglobulin E (IgE) and by promoting oxidative stress in asthma (Pelaia et al. 2020). As a consequence of intercellular contacts with mast cells, lung fibroblasts proliferate and secrete huge amounts of collagen through p38 MAPK-mediated release of IL-6 (Pelaia et al. 2021).

**Fig. 3:**
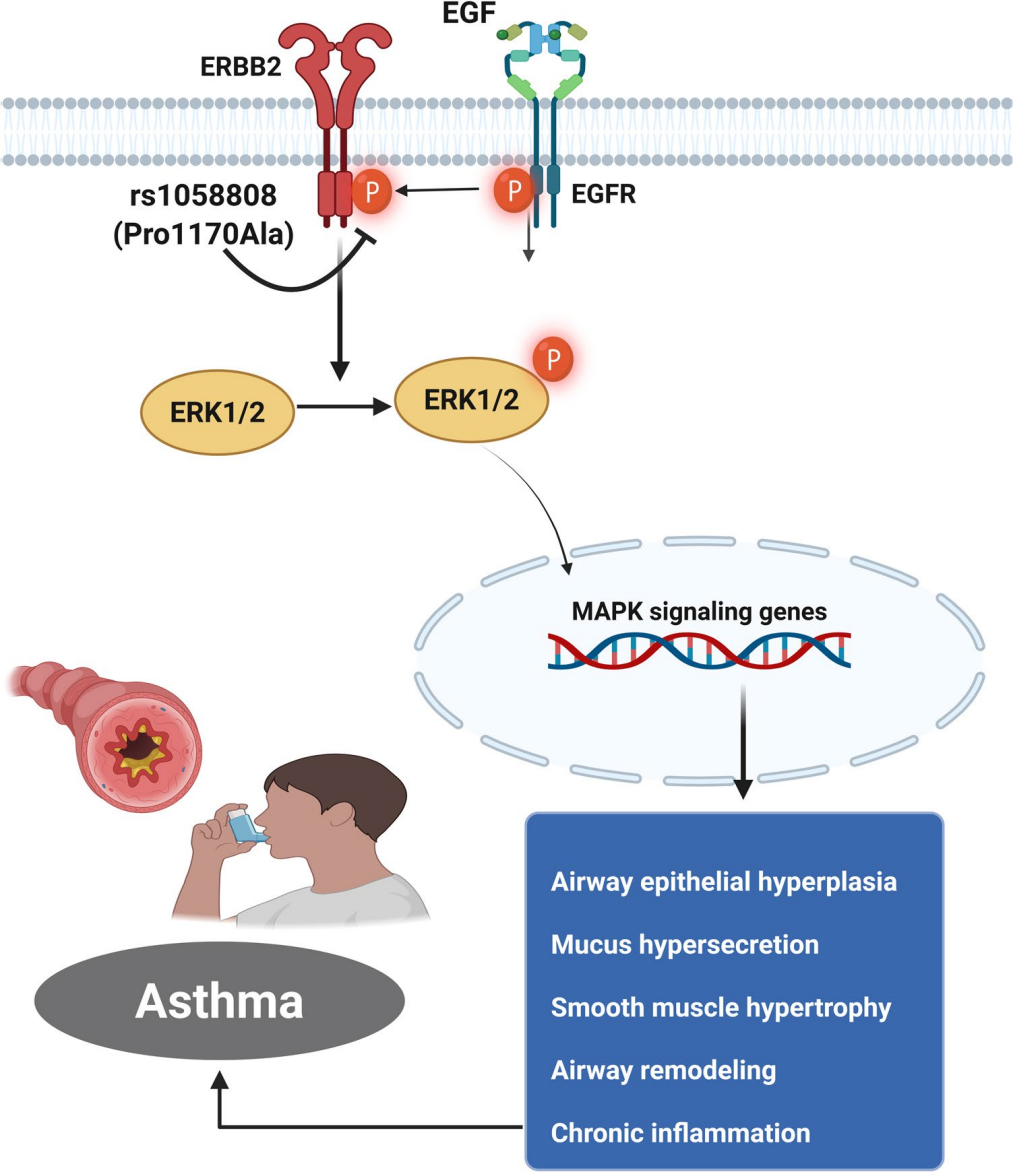
ERBB2 genetic variation modulates EGFR–ERK1/2 signaling and airway remodeling in asthma: This figure illustrates the impact of the ERBB2 single-nucleotide polymorphism rs1058808 (Pro1170Ala) on epidermal growth factor receptor (EGFR)–mediated MAPK signaling in the airway. Upon binding epidermal growth factor (EGF), EGFR undergoes phosphorylation and forms functional dimers with ERBB2, leading to downstream activation of ERK1/2. The rs1058808 (Pro1170Ala) variant in the cytoplasmic domain of ERBB2 reduces receptor phosphorylation and attenuates EGFR–ERBB2 signaling efficiency, resulting in diminished ERK1/2 activation. Reduced ERK1/2 phosphorylation alters transcription of MAPK-dependent genes involved in airway epithelial proliferation, mucus production, smooth muscle hypertrophy, and chronic inflammation. Dysregulation of this signaling axis contributes to airway remodeling and asthma pathophysiology, highlighting ERBB2–ERK1/2 signaling as a genetically influenced therapeutic target

Importantly, phospho-ERK1/2, phospho-p38α/β/γ (p-p38), and phospho-JNK1/2/3 (pJNK) immunoreactivity are found to be strongly elevated within asthmatic mice (Duan 2004). Notably, within smooth muscle cells and within the airway epithelial compartments, pERK1/2 immunoreactivity was detected. The basal compartment within columnar epithelial cells was predominantly stained for p38 phosphorylation (Duan 2004). The intensity of immunoreactivity for pERK1/2 and p-p38, as well as the numbers of airway eosinophils and neutrophils, was strongly correlated with the severity of the asthmatic model (Makled and El-Sheakh 2023). Furthermore, genetic variation in receptor tyrosine kinases can modulate downstream MAPK signaling, influencing asthma susceptibility and disease severity (Makled and El-Sheakh 2023). Of interest, ERBB2-EGFR signaling and the rs1058808 (Pro1170Ala) variant on ERK1/2 activation are implicated in airway remodeling in asthma (Sripada 2021). These findings highlighted the pathogenic role of the MAPK signaling pathway in asthma (Fig. 3).

**Fig. 4:**
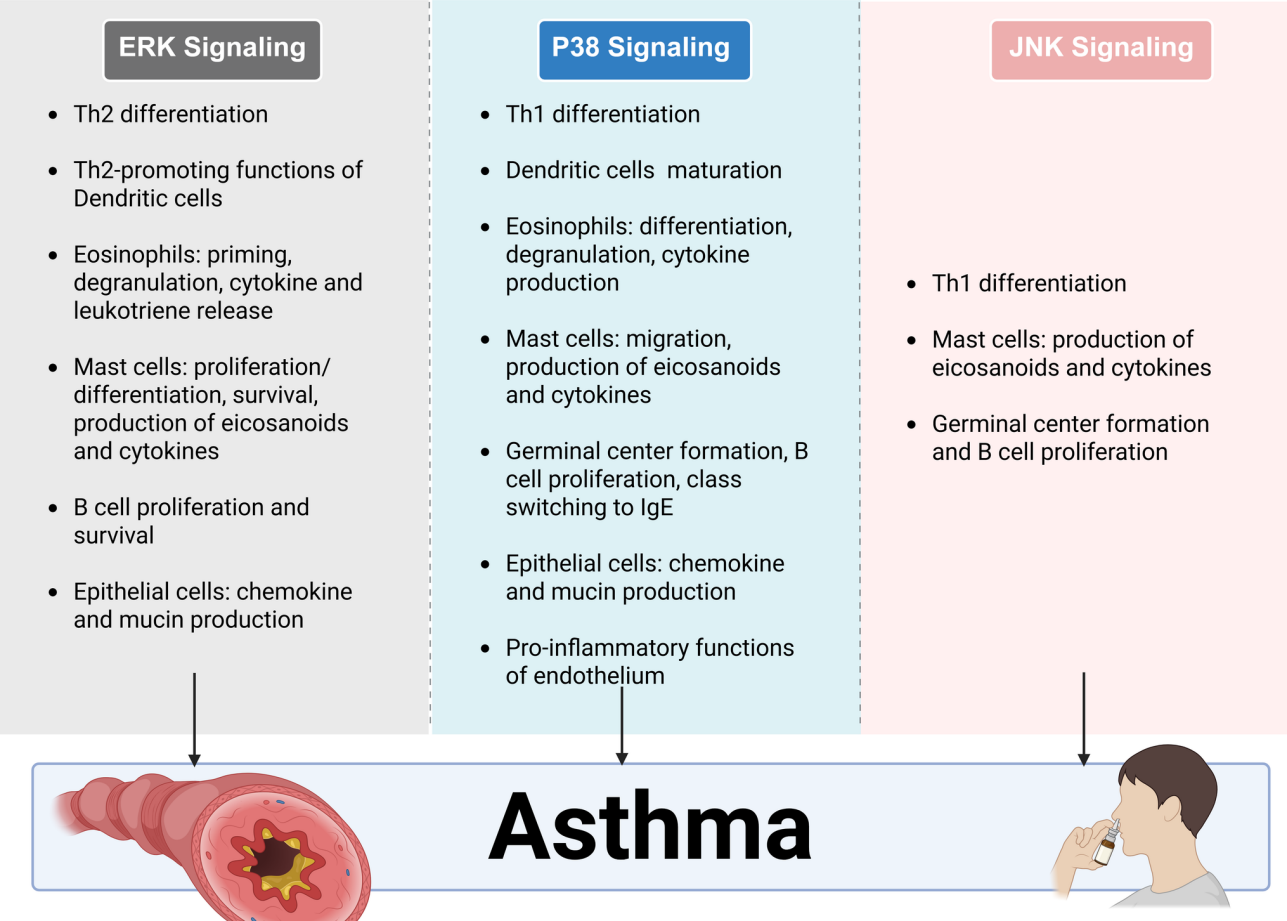
Distinct and overlapping roles of MAPK subfamilies (ERK, p38, and JNK) in asthma pathogenesis: This figure summarizes the differential contributions of the three major mitogen-activated protein kinase (MAPK) subfamilies, ERK, p38, and c-Jun N-terminal kinase (JNK), to immune regulation and airway pathology in asthma. ERK signaling predominantly promotes Th2 differentiation, dendritic cell–driven Th2 polarization, eosinophil priming and degranulation, mast cell survival and mediator release, B-cell proliferation, and epithelial chemokine and mucin production, collectively favoring allergic inflammation and airway remodeling. p38 MAPK signaling is primarily associated with Th1 differentiation, dendritic cell maturation, eosinophil activation, mast cell migration and cytokine production, germinal center formation with IgE class switching, epithelial inflammatory responses, and pro-inflammatory endothelial activation. JNK signaling contributes to Th1 differentiation, mast cell–derived cytokine and eicosanoid production, and B-cell proliferation and germinal center formation. The integrated activation of these MAPK pathways converges on chronic airway inflammation, structural remodeling, and the clinical manifestations of asthma

**Fig. 5:**
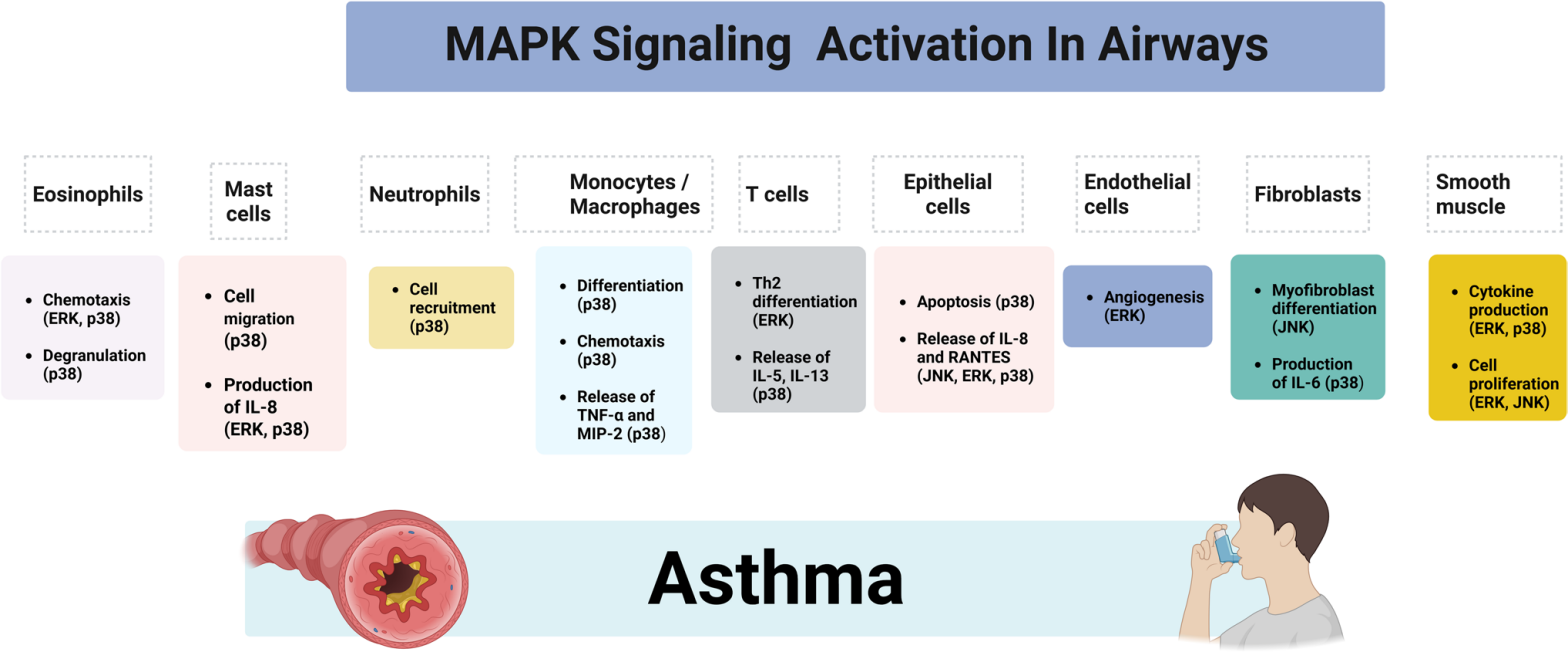
Cell-specific activation of MAPK signaling pathways in the asthmatic airway: This figure summarizes the cell-specific activation and functional consequences of mitogen-activated protein kinase (MAPK) signaling pathways within the asthmatic airway. Distinct MAPK subfamilies—ERK, p38, and JNK—are differentially engaged across immune and structural airway cells. In eosinophils, ERK and p38 regulate chemotaxis and degranulation, while mast cell migration and cytokine release are predominantly mediated by p38 and ERK signaling. Neutrophil recruitment is mainly driven by p38 activation, whereas monocytes/macrophages rely on p38 signaling for differentiation, chemotaxis, and pro-inflammatory cytokine release. In T cells, ERK promotes Th2 differentiation, while p38 contributes to IL-5 and IL-13 secretion. Airway epithelial cells integrate ERK, p38, and JNK signaling pathways to regulate apoptosis and chemokine production, including IL-8 and RANTES. Endothelial ERK signaling promotes angiogenesis, fibroblast JNK signaling drives myofibroblast differentiation, and p38-dependent IL-6 production contributes to fibrotic responses. In airway smooth muscle, ERK, p38, and JNK collectively regulate cytokine production and cell proliferation. The coordinated activation of these MAPK pathways across multiple cell types converges on airway inflammation, remodeling, and the clinical manifestations of asthma

Indeed, two ERK1/2-inducible proteins, JunB and sprouty-2 (Spry2), were also highly up-regulated in the airway tissue of asthmatic patients, with prominent JunB expression, a member of the AP-1 transcription factor complex (Sripada et al. 2021). The AP-1 complex mediates several transcriptional events induced by ERK1/2. JunB induces the Th2 cell lineage (Bahman et al. 2026). In lung morphogenesis, Spry2, a cytosolic adaptor protein, plays a fundamental role in bronchial morphogenesis and mediates receptor-induced ERK1/2 activation (Sripada et al. 2021). Another study showed that p38 MAPK phosphorylation is increased in alveolar macrophages derived from bronchoalveolar lavage fluids of asthmatic patients (Bhavsar et al. 2010). Furthermore, p38 MAPK provoked by LPS is inversely associated with corticosteroid responsiveness and positively correlated with disease severity in asthmatic models (Zeyen et al. 2022). Remarkably, an increased p38 MAPK phosphorylation in lung extracts following allergen challenge in BALB/c mice (Sultonov et al. 2022). Although ERK1/2 has emerged as a key driver of allergic airway disease, other MAPK subfamilies, including p38 and JNK, also contribute to immune regulation and airway pathology in a cell- and context-dependent manner (Reza et al. 2025). The distinct and overlapping roles of ERK, p38, and JNK signaling pathways in asthma are complex (Fig. 4**)**. Thus, allergen-induced airway inflammation can be inhibited by MAPK inhibitors, either pharmacologically or through genetic manipulation. Such progress in understanding the role of p38 MAPK in the pathobiology of asthma and COPD has led to considering p38 MAPK as a suitable molecular target for novel treatment strategies. Indeed, many studies highlighted the potential therapeutic effects of p38 MAPK inhibitors in both asthma and COPD (Kim et al. 2020; Baines et al. 2020). A cell-specific overview of MAPK pathway activation and downstream effects in the airway is provided in Fig. 5.

## Natural inhibitors of MAPK signaling pathways in asthma

Acteoside is a phenylpropanoid glycoside widely distributed in diverse herbs and has been shown to possess both anti-inflammatory and antioxidative properties across a range of disorders (Cui et al. 2018). Findings from a preclinical study revealed that acteoside improved asthmatic airway inflammation and remodeling in human bronchial epithelial cells by inhibiting ROS generation and inactivating NF-κB/MAPK pathways (Pelaia et al. 2005). In OVA-sensitized mice, acteoside significantly suppresses airway hyperresponsiveness, production of inflammatory cytokines, serum OVA-specific IgE, and TGF-β1 protein expression (Pelaia et al. 2005). Furthermore, acteoside attenuated OVA-induced oxidative stress, decreased the expression of NOX4 and iNOS protein, promoted AMPK activity, and increased the expression of antioxidant enzymes in TNF-α-induced human bronchial epithelial cells. Additionally, acteoside suppressed NF-κB and MAPK pathway activation in vivo and in vitro (Sultonov et al. 2022), suggesting that it, by reducing asthmatic airway inflammation and remodeling, may be a novel therapeutic agent for asthma management. Similarly, acteoside reduced pulmonary inflammation in asthmatic mice by enhancing the release of the anti-inflammatory cytokine from dendritic cells (Sultonov et al. 2022), indicating that acteoside displays immunomodulatory effects and plays an anti-inflammatory role in the treatment of allergic asthma.

In addition, artesunate, a semisynthetic water-soluble artemisinin derivative extracted from the plant *Artemisia annua*, can attenuate asthma airway remodeling by inhibiting the MARK pathway in BALB/c female mice (Zhang et al. 2023). Artesunate, by inhibiting the MAPK pathway signaling pathway, attenuates the overexpression found in inflammatory zone 1 (FIZZ1) in OVA-induced asthma in BALB/c female mice (Zhang et al. 2023). Of interest, artesunate can reverse glucocorticoid insensitivity by inhibiting the PI3K/AKT pathway, thereby reducing eosinophil infiltration and promoting eosinophil apoptosis in asthmatic mice (Wang et al. 2020). Additionally, artesunate regulates the expression of Fas and Bcl-2 to reduce eosinophil infiltration in lung tissue and promote eosinophil apoptosis in asthmatic mice (Wang et al. 2020). This finding provides new theoretical evidence for the pharmacological mechanism underlying artesunate’s improvement in asthma.

Moreover, the flavonoid luteolin, which has anti-inflammatory, antioxidant, and immunomodulatory effects, can inhibit inflammation in neutrophilic asthma by inhibiting IL-36γ-mediated MAPK pathways in C57BL/6 mice (Qiao et al. 2023). Also, luteolin inhibits activation of the MAPK pathway and IL-1β secretion following rhIL-36γ stimulation (Qiao et al. 2023). Interestingly, luteolin decreases the excessive production of airway mucus in asthmatic mice by inhibiting the γ-aminobutyric acid (GABA)ergic system (Qiao et al. 2023). GABA_A_ receptor (GABA_A_R) is important for promoting mucus oversecretion in lung airway epithelia. GABA_A_R, distributed in airway epithelial cells, is highly associated with mucus overproduction in respiratory diseases (Qiao et al. 2023). Additionally, luteolin reduces airway inflammation in asthma by inducing CD4^+^CD25^–^T cells to differentiate into CD4^+^CD25^+^ regulatory T cells (Qiao et al. 2023). Furthermore, luteolin can improve asthma by modulating the PI3K/Akt/mTOR signaling pathway and inhibiting autophagy in allergic asthma through inhibition of Beclin-1-PI3K catalytic domain 3 (PI3KC3) complexes (Qiao et al. 2023). Therefore, luteolin, by blocking IL-36γ-induced IL-1β expression in human bronchial epithelial cells, can reduce the secretion of inflammatory factors and lessen the inflammatory response in asthma.

gsgImportantly, ginsenosides, the primary bioactive components of *Panax ginseng*, have demonstrated significant immunomodulatory potential (Jin et al. 2023). It has been shown that ginsenosides have protective effects against the pathogenesis of asthma (Jin et al. 2023). Findings from a preclinical study demonstrated that ginsenoside Rh1 alleviates OVA/LPS-induced allergic asthma by suppressing immune cell infiltration by blocking the activation of MAPK, Akt, and NF-κB signaling pathways (Jin et al. 2023). Ginsenoside Rh1 considerably suppressed phorbol ester-induced lung inflammation and macrophage activation by overwhelming proinflammatory cytokines in A549 cells. In addition, ginsenoside Rh1 eliminated the phorbol ester-induced inflammation by inhibiting MAPK, Akt, and NF-κB p65. Furthermore, ginsenoside Rh1 inhibits eosinophil, macrophage, and neutrophil maturation through IL-4 and OVA-specific IgE production (Jin et al. 2023). Similarly, ginsenoside Rh1 attenuates OVA-induced asthma in a mouse model by regulating the balance of Th1/Th2 cytokines in serum or bronchoalveolar lavage fluid (Jin et al. 2023).

These findings highlighted that natural products that inhibit the MAPK pathways may be effective in reducing bronchial inflammation and remodeling in asthma (Table 2). Nevertheless, the animal models are not fully representative of human disease, and more animal models are needed to confirm this effect. However, the efficacy and long-term safety of natural products remain incompletely elucidated. Hence, repurposing FDA-approved drugs, such as metformin, which has well-established efficacy and safety, may be effective in treating asthma by inhibiting the MAPK signaling pathway.

**Table 2:** Targeting of the MAPK signaling pathway by natural products in asthma

Natural products	Findings	Ref.
**Acteoside**	Acteoside improved asthmatic airway inflammation and remodeling in human bronchial epithelial cells by inhibiting ROS generation and inactivating NF-κB/MAPK pathways.	(Pelaia et al. 2005)
	Acteoside improved pulmonary inflammation in asthmatic mice by enhancing the release of the anti-inflammatory cytokine from dendritic cells	(Pelaia et al. 2005
**Artesunate**	Artesunate attenuates airway remodeling in asthma by inhibiting the MARK pathway in BALB/c female mice.	(Zhang et al. 2023)
	Artesunate attenuates airway remodeling in asthma by inhibiting the MARK pathway in BALB/c female mice.	(Wang R et al. 2020)
**Luteolin**	Luteolin inhibits IL-36γ-mediated MAPK pathways in C57BL/6 mice.	(Qiao et al. 2023)
	Luteolin reduces airway inflammation in asthma by inducing CD4^+^CD25^–^T cells to differentiate into CD4^+^CD25^+^ regulatory T cells.	(Qiao et al. 2023)
	Luteolin improves asthma by modulating the PI3K/Akt/mTOR signaling pathway and inhibiting autophagy in allergic asthma through inhibition of Beclin-1-PI3K catalytic domain 3 (PI3KC3) complexes.	(Qiao et al. 2023)
**Ginsenoside Rh1**	Ginsenoside Rh1 alleviates OVA/LPS-induced allergic asthma by suppressing immune cell infiltration via blocking the activation of MAPK, Akt, and NF-κB signaling pathways.	(Jin et al. 2023)
	Ginsenoside Rh1 attenuates OVA-induced asthma in the mouse model by regulating Th1/Th2 cytokines balance in serum or bronchoalveolar lavage fluid.	(Jin et al. 2023)

## The potential role of metformin in asthma

Metformin is an insulin-sensitizing drug used in the management of type 2 diabetes (T2D) and polycystic ovary syndrome. Moreover, metformin has pleiotropic effects, including anti-inflammatory, antioxidant, antiapoptotic, and antiproliferative effects (Al-Kuraishy et al. 2023). Metformin acts by increasing cellular AMP-activated protein kinase (AMPK), thereby enhancing peripheral insulin sensitivity and suppressing hepatic gluconeogenesis and glycogenolysis (Alshehri et al. 2025). Metformin does not affect insulin secretion from pancreatic β cells; therefore, it does not cause hypoglycemia in T2D patients or healthy volunteers (Al-Kuraishy et al. 2023). Chemically, metformin is a hydrophilic base that exists as a cationic species at physiological pH (Al-Kuraishy et al. 2020). Therefore, its passive diffusion across the cell membrane is limited. Metformin is mainly absorbed from the small intestine via organic cation transporters (OCTs), which also mediate its hepatic uptake and renal excretion (Al-Qahtani et al. 2024). The bioavailability of metformin is 40–60%, the liver does not metabolize it, and it is excreted unchanged by the kidney (Alameen et al. 2025).

Metformin has been shown to reduce eosinophilic airway inflammation in obese mice, normalizing TNF-α levels in bronchoalveolar lavage and restoring AMPK levels in male C57BL6/J mice (Calixto et al. 2013). Metformin reduced NF-κB subunit p65 binding to the iNOS promoter in lung tissue from obese mice. Lower levels of phosphorylated AMPK and its downstream target, acetyl-CoA carboxylase (ACC), were found in the lung tissue of obese mice, which were restored by metformin (Calixto et al. 2013). Metformin may also inhibit airway smooth muscle cell proliferation through AMPK-dependent pathways (Pan et al. 2018). Activation of AMPK slightly attenuated TGF-β1-induced miR-206 suppression, but dramatically suppressed TGF-β1-induced HDAC4 upregulation and significantly increased HDAC4 phosphorylation, ultimately leading to a reduction in upregulated cyclin D1 protein expression (Pan et al. 2018). Thus, activation of AMPK modulates the miR-206/HDAC4/cyclin D1 signaling pathway, chiefly targeting HDAC4, to suppress airway smooth muscle cell proliferation and, therefore, has potential value in the prevention and treatment of asthma by alleviating airway remodeling. Also, metformin alleviates airway inflammation in obese asthma through regulating metabolic parameters and immune response (Guo et al. 2021). Metformin reversed the obese state and alleviated airway inflammation and remodeling, with increased Tregs and related transcription factors in HFD- and OVA-induced airway sensitization (Al-Qahtani et al. 2024), suggesting that the anti-inflammatory effect of metformin may be mediated by increasing Tregs. Similarly, metformin, via AMPK activation, reduces NLRP3/caspase-1 expression and the release of inflammatory cytokines, thereby attenuating airway inflammation in obese asthma mice (Xing et al. 2025). Liu et al. demonstrated that metformin reduces airway inflammation, mucus hypersecretion, eosinophil infiltration, and IL-5 and IL-13 levels in bronchoalveolar lavage fluid. Metformin effectively reduces airway inflammation in acute allergic asthma, and its protective role may be mediated by suppressing ILC2s, suggesting that metformin may be a potential prophylactic candidate for preventing the transition from subclinical inflammation to overt allergic asthma (Liu et al. 2025).

Notably, oxidative stress is consistently implicated in the pathophysiology of asthma in the current literature, despite controversies over whether it is a consequence of inflammation or a contributing factor (Park et al. 2012). A case–control study revealed that patients with uncontrolled asthma have a high degree of ROS formation, causing considerable oxidative stress, and increased MDA level, SOD activity, and reduced GPx activity were predictors of poorly controlled asthma (Park et al. 2012). The molecular pathway by which oxidative stress exerts its effects on ASM in asthma involves the Nrf2/HO-1, MAPK, and PI3K/Akt pathways, which may act by connecting to various upstream and downstream signaling molecules and contribute to the amplification of airway hyperresponsiveness (Park et al. 2012). Moreover, NADPH oxidases, which induce oxidative stress, are reduced by metformin, which decreases oxidative stress in lung tissues by activating the AMPK signaling pathway (Song and Zou 2012).

**Fig. 6:**
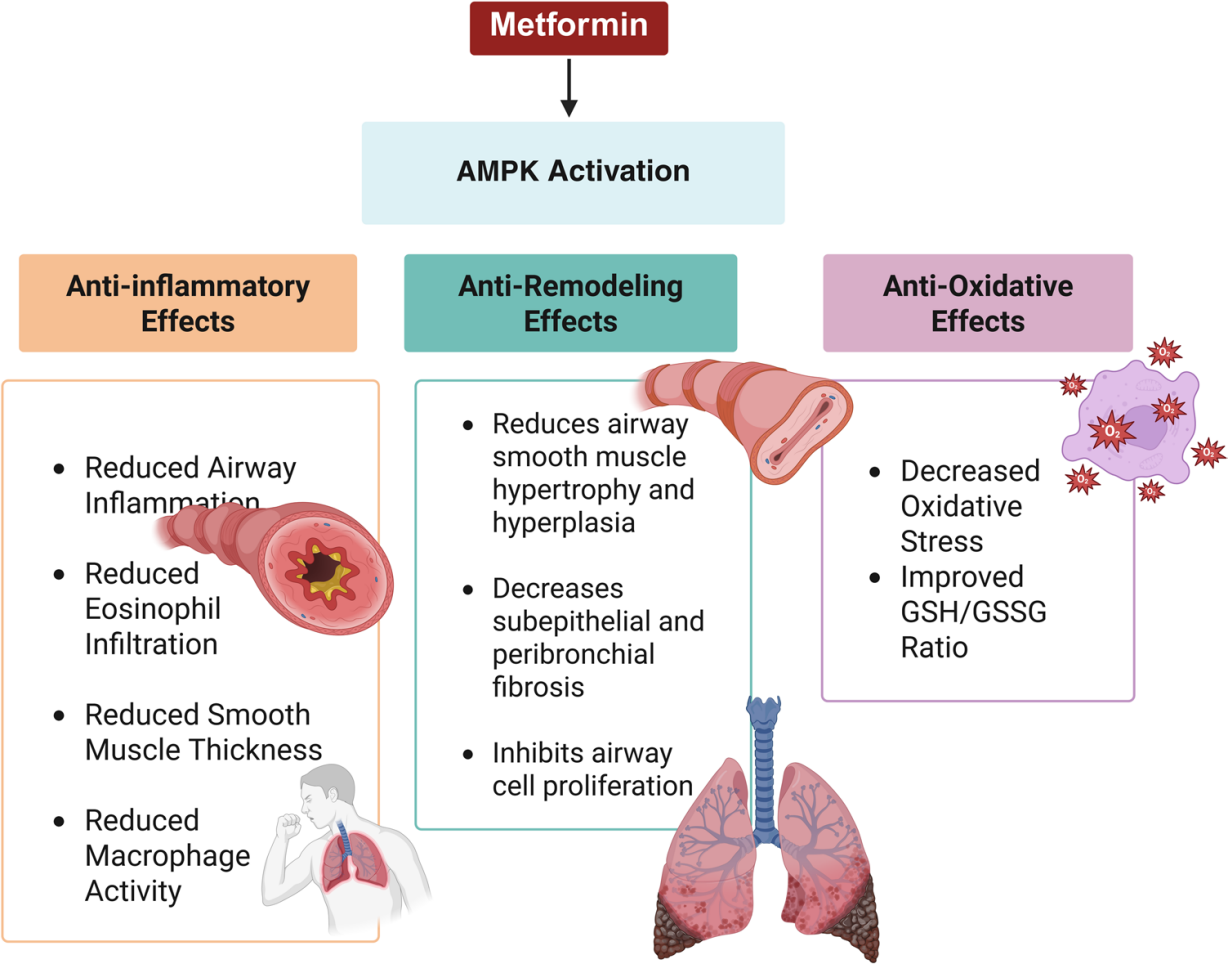
Metformin-mediated AMPK activation attenuates airway inflammation, remodeling, and oxidative stress in asthma: This schematic illustrates the proposed mechanisms by which metformin exerts protective effects in asthma by activating AMP-activated protein kinase (AMPK). Metformin-induced AMPK activation leads to broad anti-inflammatory effects, including reduced airway inflammation, decreased eosinophilic infiltration, suppression of macrophage activity, and attenuation of airway smooth muscle thickening. In parallel, AMPK activation limits airway remodeling by reducing smooth muscle hypertrophy and hyperplasia, decreasing subepithelial and peribronchial fibrosis, and inhibiting airway cell proliferation. Additionally, metformin enhances anti-oxidant defenses by reducing oxidative stress and improving the glutathione redox balance, as reflected by an increased GSH/GSSG ratio. Collectively, these interconnected actions position metformin as a promising repositioned therapeutic agent targeting metabolic–inflammatory signaling networks in asthma

Furthermore, metformin inhibits TGF-β-induced NOX4 expression, which induces ROS generation and myofibroblast differentiation (Song and Zou 2012). Thus, AMPK may be an important target for the development of new drugs to treat airway inflammation and remodeling in bronchial asthma. The proposed mechanisms by which metformin activates AMPK to exert anti-inflammatory, anti-remodeling, and antioxidant effects are summarized in Fig. 6.

These verdicts highlighted the possible therapeutic efficacy of metformin in mitigating airway inflammation, oxidative stress, and remodeling in asthma. However, the effect of metformin on the MAPK signaling pathway in asthma is limited. Nevertheless, metformin, by activating AMPK signaling, can reduce p38 MAP kinase activity, thereby inhibiting IL-6 expression and gut inflammation (Fusco et al. 2018). Notably, gut dysbiosis is implicated in chronic inflammatory respiratory disorders, particularly asthma (Frati et al. 2018). The role of the microbiota in a healthy immune response is generally acknowledged, and gut dysbiosis may contribute to chronic inflammatory respiratory disorders, particularly asthma. Further investigations are needed to improve our understanding of the role of the microbiome in inflammation and its influence on important risk factors for asthma, including tobacco smoke and host genetic features. Numerous studies have established that inflammation in atopic asthma is linked to the composition of the microbiota and appears associated with the severity of airway obstruction, and that treatment with inhaled corticosteroids is associated with changes in the airway inflammatory response to the microbiota (Wang et al. 2024; Du et al. 2022). Findings from a preclinical study observed that tryptophan metabolites improve asthma symptoms in mice and reduce inflammatory cells in lung tissues, especially indole-3-carbaldehyde (Park et al. 2012; Song and Zou 2012), suggesting that tryptophan metabolites may modulate asthma through the gut microbiota, offering potential benefits for clinical asthma management. In this state, metformin treatment effectively restored serum levels of tryptophan metabolites and several aryl hydrocarbon receptor ligands in diabetic mice (Wang et al. 2024; Du et al. 2022). Therefore, metformin, by modulating gut tryptophan metabolites, can improve airway inflammation in asthma.

Moreover, mast cell activation and anaphylaxis are negatively regulated by AMPK, which is downregulated by ERK1/2 in mast cells (Zheng et al. 2025). Zingerone, a primary bioactive compound in ginger, demonstrates protective effects in asthma via AMPK activation. Zingerone, both *in vivo* and *in vitro,* inhibits Akt, ERK1/2, JNK, p38, and the release of proinflammatory cytokine via LKB1/AMPK activation (Zheng et al. 2025). Therefore, metformin-induced AMPK activation can attenuate ERK1/2-mediated mast cell activation in asthma. Correspondingly, metformin hampers mast cell activation and airway resistance in asthmatic diabetic rats (Fu et al. 2023). Moreover, metformin downregulated IR and IGF-1R via DNA methyltransferase 1-dependent methylation, thereby repressing mast cell activation and airway resistance. Thus, DNA methyltransferase 1 may be a metformin target gene (Fu et al. 2023). However, a retrospective study highlighted that long-term use of metformin was associated with a higher risk of chronic urticaria in T2D patients (Yen et al. 2022).

Therefore, metformin, by regulating MAPK-ERK1/2 signaling in the lung and airways, can reduce the severity and complications of asthma.

## Discussion

The pathogenesis of asthma is linked to the exaggeration of airway MAPK-ERK1/2 signaling. This review has covered the strong evidence linking ERK1/2 hyperactivity to the severity of clinical asthma, its involvement in several cellular processes in the airway, and the potential therapeutic benefits of regulating it. A key component of the case for focusing on this pathway is the consistent finding of increased pERK1/2 in the smooth muscle and airway epithelium in asthma, which correlates with disease severity and the infiltration of inflammatory cells (Xie et al. 2007). Functional investigations in mouse models have demonstrated that pharmacological suppression of MEK1/2, the direct upstream activator of ERK1/2, significantly attenuates airway inflammation, hyperresponsiveness, and key remodeling characteristics (Liu et al. 2008). This places the Raf/MEK/ERK axis as a convergent signaling pathway activated by a wide array of asthma-relevant stimuli, including growth factors, oxidative stress, and allergens, via IgE and cytokine receptors (Zhang, et al. 2025). Activation of ERK1/2 exerts several pleiotropic downstream effects, including the induction of proinflammatory cytokine expression, such as IL-1β and IL-6, the enhancement of Th2 cell differentiation through transcription factors, and the promotion of the growth and survival of structural airway cells, including smooth muscle and fibroblasts (Zheng et al. 2025). Given its concurrent actions on the immune response and structural remodeling, both critical, interrelated pillars of asthma pathogenesis, ERK1/2 is a particularly attractive target.

However, the biological complexity of the system and the heterogeneity of the disease make the ERK1/2 in asthma more complicated. Members of the MAPK family, including JNK and p38, often act in a coordinated, context-dependent manner. JNK is important in the smooth muscle of healthy controls (Zhu et al. 2025), while p38 is restricted to the basal epithelial layer (Qu et al. 2023). This suggests different roles for each branch: p38 may regulate stress responses; JNK may have homeostatic functions that are disrupted in disease; and ERK1/2 may be more critical for orchestrating the allergic inflammatory response and epithelial-mesenchymal communication. Even though its role in asthma appears largely pathogenic, global and systemic inhibition may disrupt immunological homeostasis or essential repair mechanisms, with unexpected consequences. This underscores the importance of targeted therapeutic strategies over blanket inhibition of the core kinase, such as focusing on specific upstream activators, specific cell types (e.g., T cells or airway smooth muscle), or specific downstream effectors.

Genetic data, such as the Pro1170Ala mutation in ErbB2 (HER2), which is associated with reduced ERK1/2 phosphorylation and protection against asthma (Kim et al. 2020), strongly supports the importance of the pathway. Hence, consideration of pharmacologic modulators, such as metformin, is consistent with this genetic observation. Investigating the anti-asthmatic properties of metformin, at least partly mediated by AMPK activation, introduces an important layer of interaction between metabolic and inflammatory signaling pathways. The activation of AMPK is usually anti-inflammatory and inhibits cell growth, acting as a physiological counter-regulator to pathways such as mTOR (Calixto et al. 2013). The fact that metformin reduced oxidative stress, fibrosis, and eosinophilia in models of chronic asthma suggests that ERK1/2 signaling is indirectly dampened (Zheng et al. 2025). A possibility is the suppression of growth factor receptor signaling or the inhibition of Raf by AMPK. This is a treatment approach that extends beyond the use of direct kinase inhibitors: the use of pharmacological agents that generally enhance cellular homeostasis, with modification of ERK1/2 activity as one beneficial consequence.

The main challenges for the therapeutic use of ERK1/2 pathway inhibitors are safety and selectivity. MEK inhibitors have several adverse effects, including cardiac, ophthalmic, and dermatological toxicities, which are intolerable for a chronic illness such as asthma (Garutti et al. 2022; Jeng-Miller et al. 2024). Therefore, more sophisticated methods are likely to be the future. The development of inhaled MEK/ERK inhibitor formulations is one strategy to minimize systemic exposure and off-target effects while achieving high local lung concentrations. Identifying essential, asthma-specific upstream receptors or adaptor proteins, such as sprouty-2, that provide a more limited treatment window is another approach. In addition, patient selection based on biomarkers will be crucial. A potential application could be in severe, eosinophilic, or neutrophilic asthma phenotypes, where the pathway is most active, given the association between pERK1/2 immunostaining and disease severity. Those expected to respond to such targeted therapy may be stratified by measuring pERK1/2 levels in bronchial biopsies or, possibly, in surrogate peripheral cells (Roux and Blenis 2004; Wang et al. 2004).

Without considering ERK1/2 in relation to current asthma treatments, the discussion would not be complete. Indeed, inhibition of ERK1/2 activation and resulting AP-1-driven gene transcription may be one component of the benefits of asthma. Persistent activation of the MAPK pathways, particularly p38, which can phosphorylate and inactivate glucocorticoid receptors, has been associated with corticosteroid resistance, a major clinical problem in asthma (Sevilla et al. 2021). This would suggest that in resistant situations, add-on therapy with an inhibitor of the MAPK pathway could restore steroid sensitivity. Preclinical and clinical studies are needed to demonstrate whether an improved anti-inflammatory benefit with reduced corticosteroid-associated side effects can be achieved by combining a low dose of a MEK inhibitor with inhaled corticosteroids in asthma (Bhavsar et al. 2010).

Ultimately, this review highlights several key directions for future research. First, conditional, cell-specific deletion models will be required to resolve the exact contributions of ERK1 versus ERK2 isoforms. Although both are pharmacologically inhibited, genetic studies suggest that their functions may not overlap. Second, the function of ERK1/2 in nonallergic asthma phenotypes, such as neutrophilic or obesity-related asthma, remains unknown; therefore, additional studies will be required. Third, to understand the larger signaling network, a comprehensive mapping of the interaction between ERK1/2 and other pathways in asthma, including the HIF-VEGF axis, Wnt/β-catenin, and autophagy, will be required. Hence, medication repurposing, such as metformin for asthma, represents a near-term opportunity for clinical trials, especially in patients with metabolic comorbidities, providing a useful test of the concept that modulation of these intracellular pathways may result in therapeutic benefit. Now at issue is teasing biological complexity to develop safe, effective, and targeted modulation strategies. Biomarker-driven individualized therapy will need to be rooted in a fundamental understanding of asthma phenotypes. Such studies move closer to the goal of precision therapy for asthma by targeting intracellular nodes, such as ERK1/2, rather than broad anti-inflammatory agents, and hold promise for improved management of severe and therapy-resistant forms of this prevalent and debilitating disease. The next frontier in the ongoing campaign to reduce the global burden of asthma involves adopting network-modulating drugs, such as metformin, or adding pathway-specific inhibitors to existing therapies.

## Limitations and future directions

Despite the substantial experimental and translational evidence supporting the involvement of MAPK–ERK1/2 signaling in asthma pathophysiology, several important limitations should be acknowledged. First, the majority of mechanistic insights discussed in this review are derived from pre-clinical models and ex vivo human tissue studies, which, although informative, may not fully recapitulate the complexity and heterogeneity of human asthma. As well, ERK1/2 signaling may not play an equivalent pathogenic role across all asthma subgroups. Second, most pharmacological studies targeting the ERK1/2 pathway rely on upstream MEK inhibitors, which lack isoform specificity and often inhibit both ERK1 and ERK2 simultaneously. This limits the ability to delineate the distinct and potentially non-redundant roles of ERK1 versus ERK2 in immune regulation, airway remodeling, and epithelial repair. Moreover, systemic inhibition of the ERK pathway raises significant safety and tolerability concerns, particularly for a chronic disease such as asthma, where long-term therapy is required. Third, although indirect modulation of ERK1/2 signaling via AMPK and metformin repositioning is an attractive strategy, the current evidence base remains largely indirect and associative. The precise molecular mechanisms linking AMPK activation to ERK1/2 suppression in specific airway cell types remain incompletely defined, and clinical data supporting metformin’s efficacy in asthma are limited and confounded by metabolic comorbidities. Looking forward, several key research directions are required to translate ERK1/2-targeted strategies into clinically viable therapies. Cell-specific and isoform-specific genetic models are urgently needed to clarify the relative contributions of ERK1 and ERK2 in immune cells, airway epithelium, and airway smooth muscle. Such studies would inform the rational design of more selective pharmacological modulators with improved safety profiles. In parallel, the development of lung-targeted delivery systems, including inhaled MEK or ERK inhibitors, represents a promising approach to minimize systemic exposure while achieving therapeutically relevant concentrations in the airway. Advances in nanotechnology and inhalation pharmacology may facilitate this strategy and warrant further investigation.

Another critical future direction lies in integrating biomarker-driven precision medicine approaches. Biomarkers such as tissue or peripheral phospho-ERK1/2 expression, downstream transcriptional signatures, or pathway-specific cytokine profiles could enable patient stratification and identify asthma phenotypes most likely to benefit from ERK1/2-modulating therapies. This is particularly relevant for severe, corticosteroid-resistant asthma, where unmet therapeutic needs remain substantial. Ultimately, future clinical research should investigate combination strategies, including modulation of the ERK1/2 pathway as an adjunct to inhaled corticosteroids or biologics, to restore steroid sensitivity or attenuate airway remodeling. Well-designed clinical trials, particularly in patients with severe or metabolically dysregulated asthma, will be crucial in determining whether modulation of intracellular signaling nodes, such as ERK1/2, can deliver a durable clinical benefit.

## Conclusion

The MAPK-ERK1/2 signaling pathway plays a major role in the inflammatory and remodeling phases of asthma pathophysiology. The potential for its inhibition in pre-clinical studies, along with the correlation of its hyper-activation with the severity of the condition, makes this potential even more important. The possibility of using the regulatory pathway of this mechanism with an indirect modulator, such as metformin, further enhances this potential. However, the hurdles of safety, specificity, and the varying nature of asthma conditions must be overcome to realize this potential.

The next generation of studies should aim to develop inhalational, lung-targeting inhibitors. The validation of biomarkers, such as tissue phospho-ERK1/2 expression, is also required to help distinguish the patient phenotype most likely to respond, especially in refractory, profound, or repair-dominant asthmatic conditions. To facilitate targeted therapy, it is also crucial for basic scientific studies to clarify, through conditional genetics, the varying roles of the ERK1 isoform versus the ERK2 isoform within individual airway cells. Another approach could be the investigation of combinations, helping to identify if there is a potential for inhibiting the ERK1/2 signaling pathway, possibly making quiescent asthmatics amenable again to treatment by systemic corticosteroids, or by targeting indirect modulators such as metformin, being already off-shelf, perfectly safe, effective, cheap, scanning every aspect, helping asthmatics towards targeted personal approach by scientific, step-by-step methodologies, starting from drug distribution up to new targets and combinations.

## Data Availability

This manuscript does not report data generation or analysis.
